# Maternal *Streptococcus agalactiae* colonization in Europe: data from the multi-center DEVANI study

**DOI:** 10.1007/s15010-024-02380-0

**Published:** 2024-09-08

**Authors:** Florens Lohrmann, Androulla Efstratiou, Uffe B. Skov Sørensen, Roberta Creti, Antoaneta Decheva, Pavla Křížová, Jana Kozáková, Javier Rodriguez-Granger, Manuel De La Rosa Fraile, Immaculada Margarit, Daniela Rinaudo, Domenico Maione, John Telford, Graziella Orefici, Mogens Kilian, Baharak Afshar, Pierrette Melin, Reinhard Berner, Markus Hufnagel, Mirjam Kunze, DEVANI Study Group, K Poulsen, L Karstens, L Baldassarri, F Rigat, A Berardi, M Imperi

**Affiliations:** 1 Department of Pediatrics and Adolescent Medicine, University Medical Center, Medical Faculty, University of Freiburg, Freiburg, Germany; 2 UK Health Security Agency, London, UK; 3 Department of Biomedicine, Health, Aarhus University, Aarhus, Denmark; 4 Department of Infectious Diseases, Istituto Superiore Di Sanità, Rome, Italy; 5 National Center of Infectious and Parasitic Diseases, Sofia, Bulgaria; 6 National Institute of Public Health, Prague, Czech Republic; 7 Servicio Andaluz de Salud, Hospital Universitario Virgen de Las Nieves, Granada, Spain; 8 GSK Vaccines, Siena, Italy; 9 Department of Clinical Microbiology, National Reference Center Streptococcus Agalactiae, University Hospital Center of Liege, Liege, Belgium; 10 Department of Pediatrics, University Hospital Carl Gustav Carus, Technische Universität Dresden, Dresden, Germany; 11 Department of Obstetrics and Gynecology, University Medical Center, Medical Faculty, University of Freiburg, Freiburg, Germany

**Keywords:** Group B streptococcus, *Streptococcus agalactiae*, Maternal colonization, Neonatal sepsis, Early-onset disease, Vertical transmission, Intrapartum prophylaxis, GBS vaccine

## Abstract

**Introduction:**

Despite national guidelines and use of intrapartum antibiotic prophylaxis (IAP), *Streptococcus agalactiae* (group B streptococci (GBS)) is still a leading cause of morbidity and mortality in newborns in Europe and the United States. The European DEVANI (Design of a Vaccine Against Neonatal Infections) program assessed the neonatal GBS infection burden in Europe, the clinical characteristics of colonized women and microbiological data of GBS strains in colonized women and their infants with early-onset disease (EOD).

**Methods:**

Overall, 1083 pregnant women with a GBS-positive culture result from eight European countries were included in the study. Clinical obstetrical information was collected by a standardized questionnaire. GBS strains were characterized by serological and molecular methods.

**Results:**

Among GBS carriers included in this study after testing positive for GBS by vaginal or recto-vaginal sampling, 13.4% had at least one additional obstetrical risk factor for EOD. The five most common capsular types (i.e., Ia, Ib, II, III and V) comprised ~ 93% of GBS carried. Of the colonized women, 77.8% received any IAP, and in 49.5% the IAP was considered appropriate. In our cohort, nine neonates presented with GBS early-onset disease (EOD) with significant regional heterogeneity.

**Conclusions:**

Screening methods and IAP rates need to be harmonized across Europe in order to reduce the rates of EOD. The epidemiological data from eight different European countries provides important information for the development of a successful GBS vaccine.

**Supplementary Information:**

The online version contains supplementary material available at 10.1007/s15010-024-02380-0.

## Introduction

*Streptococcus agalactiae* or Group B streptococci (GBS) is a leading cause of neonatal morbidity and mortality in Europe and the United States [1, 2]. Early-onset GBS disease (EOD) in neonates due to vertical maternal transmission presents with sepsis during the first six days of life, while late-onset disease (LOD) affects infants between one week and three months of life [3–5]. The primary risk factor for EOD is maternal colonization with GBS [3, 5]. After implementation of screening guidelines and introduction of intrapartum antibiotic prophylaxis (IAP), the rate of EOD decreased from 1.7 to 0.34 per 1,000 live births in the United States [6]. After the Centers for Disease Control and Prevention (CDC) guidelines for the prevention of GBS disease were released in 2002, multiple European countries adopted universal screening for GBS colonization of pregnant women and intrapartum antibiotic prophylaxis (IAP) in case of GBS colonization [7–9]. However, some European countries, e.g., the United Kingdom (UK) follow a risk-based approach. The Royal College of Obstetricians and Gynaecologists (RCOG) recommends IAP to women in labour with one of the following risk factors: prolonged premature rupture of membranes (PROM) ≥ 18 h, prematurity, GBS bacteriuria during the current pregnancy, previous delivery of an infant with invasive GBS disease, intrapartum fever ≥ 38 °C or preterm delivery [10]. The reported rate of GBS colonization in most European countries ranges from 4 to 36%, with most studies reporting rates higher than 30% [10–14].

The pan-European DEVANI (Design of a Vaccine Against Neonatal Infections) consortium was established in 2008 with three main objectives. First, to assess disease burden and GBS capsular sero-/genotype distributions to facilitate the design of a GBS vaccine. Second, to standardize diagnosis of maternal colonization and neonatal infection. Third, to improve vaccine design by investigating naturally acquired antibody responses in pregnant women. Next to the already published parts of the DEVANI study [5, 15–21], we here report the clinical and microbiological characteristics of 1,083 pregnant women colonized with GBS and of nine mother-infant pairs with EOD cases.

Despite substantial efforts to reduce the disease burden in neonates, GBS remains the leading pathogen in the vulnerable neonatal population. Thus, the development of a multi-valent GBS vaccine for pregnant women has received increasing attention [22, 23]. Several phase-II studies paved the way for a broader application [24, 25]. Detailed knowledge of prevalence is required to tailor vaccine coverage of the circulating capsular serotypes. This study enhances the understanding of the variation in GBS serotype prevalence across different geographical regions and over time.

## Material and methods

### Study participants

Eight European countries cooperated within the DEVANI consortium (Belgium, Bulgaria, Czech Republic, Denmark, Germany, Italy, Spain, and the United Kingdom), together with Novartis Vaccines, Siena, Italy. The study was approved by the Institutional Review Board Committee of each participating institution. All enrolled women and parents of infected neonates signed a consent form. All GBS isolates were obtained during the DEVANI project between 2008 and 2010. Maternal and neonatal clinical data were extracted from the patient charts through a standardized questionnaire. Not all items were available from every single study participant, explaining the different denominators reported throughout the manuscript. For data capture and management, a secure online platform was available to all participants involved in the project.

### Maternal GBS colonization

Lower recto-vaginal swabs and blood samples were prospectively collected from healthy pregnant women using standardized methods. Prenatal screening was recommended in all participating centers even in countries where it is not recommended by official guidelines. Maternal GBS colonization was defined as GBS isolation by culture from either vagina, rectum or perianal region before delivery [14, 16, 17].

### Laboratory procedures and GBS strain characterization

Detailed microbiologic procedures were previously published [16, 17]. Briefly, capsular serotyping of GBS strains was performed by standardized latex agglutination tests (Immulex kit; SSI, Denmark; now commercialized by SSI diagnostica). Capsular and pilus genotypes were determined by polymerase chain reaction (PCR), as described previously [16, 17, 20, 26]. Of note, Fig. 2a and supplemental Tables 1, 2 and 3 display the results for capsular serotyping, unless the isolate was untypeable by serotyping, in these cases the capsular genotype is used for the analyses. Pilus surface expression was assessed by flow cytometry using monoclonal antibodies raised against pilus 1, 2a and 2b proteins. In addition, multi-locus sequence typing (MLST) of GBS isolates was performed [16, 17].

### Intrapartum antibiotic prophylaxis

IAP was defined as antibiotics given at any time during labor for the prevention of EOD in GBS-colonized pregnant women [14]. Appropriate IAP was defined as targeted intravenous antibiotics of at least two doses for at least four hours prior to delivery. The agent of choice is penicillin or ampicillin. First-generation cephalosporins are recommended for women with a history of penicillin allergy [10, 27]. Clindamycin was equally considered “appropriate”, despite substantial rates of antibiotic resistance. For our analysis, “inadequate IAP” was any IAP not following the criteria for “appropriate IAP”.

### Early-onset invasive neonatal GBS disease

EOD was defined as GBS positive blood or cerebrospinal fluid culture in the first 6 days of life [14, 28].

### Statistical methods

**Fig. 1:**
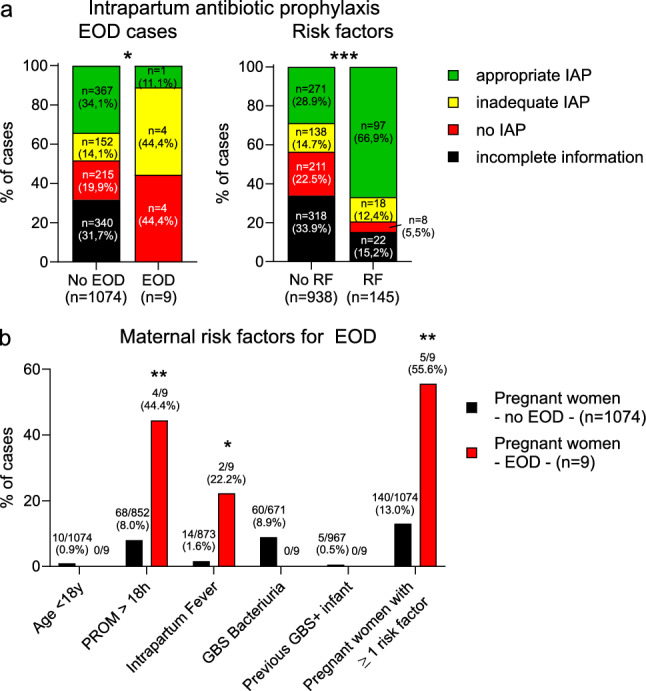
Intrapartum antibiotic prophylaxis to colonized pregnant women and maternal risk factors for EOD. **A** Appropriate, inadequate or no IAP given to colonized pregnant women. Left, comparing mothers giving birth to infants without EOD (“no EOD”) or infants who developed EOD (“EOD”). P-value using Fisher’s exact test comparing appropriate versus inadequate or no IAP is 0.038. Right, comparing mothers with or without additional risk factors. P-value using Fisher’s exact test comparing appropriate versus inadequate or no IAP is < 0.001. **B** Maternal risk factors for giving birth to infants with EOD, stratified into mothers who gave birth to infants without (“no EOD”) or with EOD (“EOD”). P-values using Fisher’s exact test are 0.004 for PROM > 18 h; 0.01 for intrapartum fever; and 0.003 for any risk factor. *EOD* early onset disease. *IAP* intrapartum antibiotic prophylaxis. *PROM* premature rupture of membranes

**Fig. 2:**
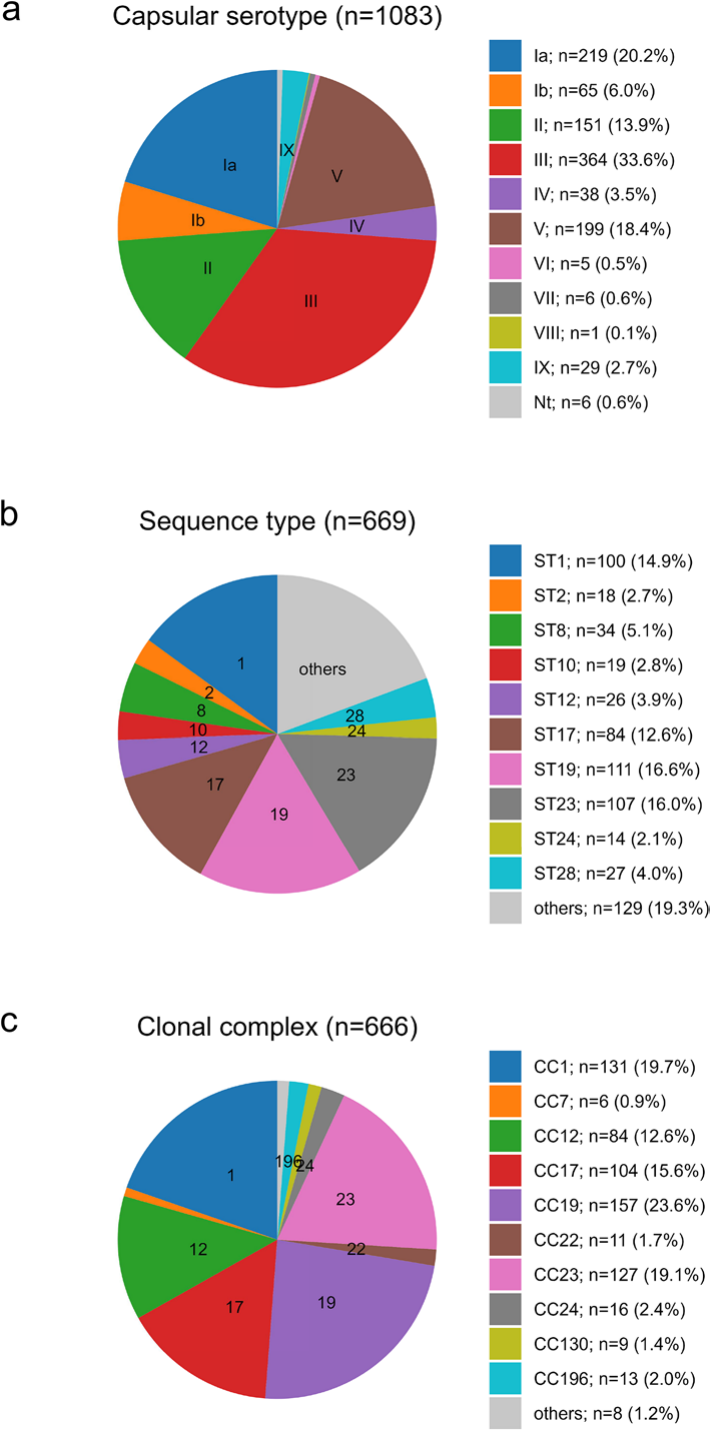
Distribution of GBS capsular serotypes, clonal complexes and sequence types. **A** Distribution of capsular serotypes of 1083 GBS isolates from colonized pregnant women. Results from capsular serotyping are displayed, unless the isolate was untypeable (112 isolates), in that case the capsular genotype is used. All ten detected serotypes are displayed. **B** Distribution of multi-locus sequence types of 669 GBS isolates from colonized pregnant women. The ten most abundant sequence types are displayed, “others” contain 73 additional STs. C. Distribution of clonal complexes of 666 GBS isolates from colonized pregnant women. The ten most abundant clonal complexes are displayed, “others” contain three additional CCs. *Nt* not typable

**Fig. 3:**
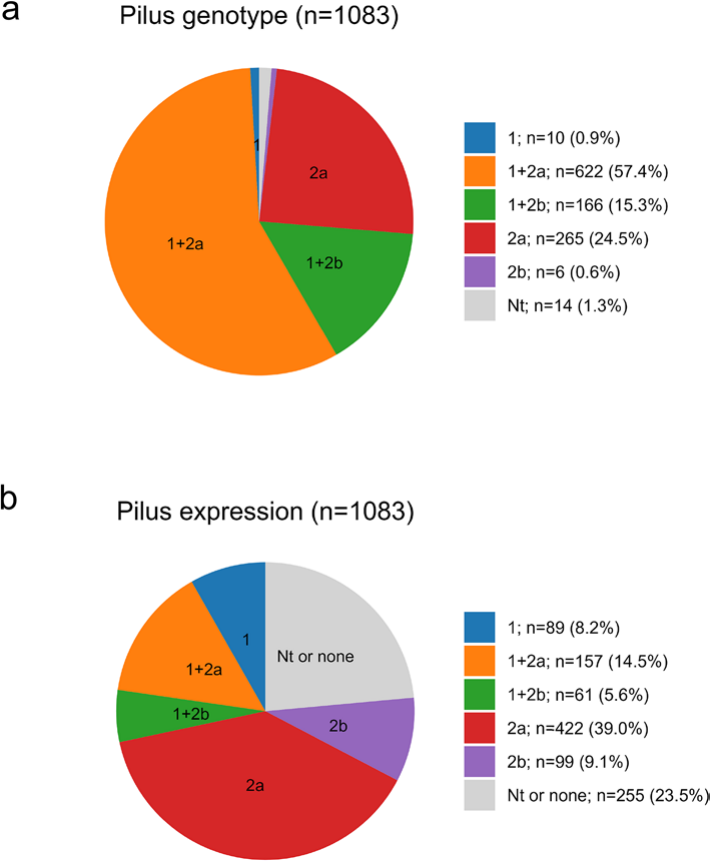
Distribution of GBS pilus genotype and pilus expression. **A** Distribution of pilus genotypes of GBS isolates from colonized pregnant women. **B** Distribution of pilus expression of GBS isolates from colonized pregnant women. *EOD* early onset disease. *Nt* not typable

Differences in rates of IAP, stratified according to EOD or the presence of risk factors (Fig. 1a) were calculated using Fisher’s exact test. Similarly, the association between additional maternal risk factors and giving birth to a neonate with EOD (Fig. 1b) was analyzed using Fisher’s exact test. The figure legends indicate the exact p-values. Supplemental Tables 1, 2 and 3 list “raw p-values”, calculated by either chi square, or Fisher’s exact test if any of the categories had a number of five or below. Benjamini–Hochberg adjustments were calculated to reduce the risk of type-I errors and listed as “adjusted p-values”. Statistical analyses were performed and figures were generated either with GraphPad Prism (Fig. 1 and Fig. S1) or using R [29], version 4.3.3 (Figs. 2 and 3 and supplemental Tables 1, 2, 3, 4).

## Results

Overall, 1083 pregnant women (PW) with a GBS-positive culture result were included in the study. The numbers of PW enrolled from the different countries were: Belgium 111, Bulgaria 29, Czech Republic 107, Denmark 82, Germany 363, Italy 93, Spain 187, and the UK 111, respectively. Supplemental Table 1 lists the capsular serotype distribution across countries.

### Clinical characteristics

Table 1 presents the maternal demographics and clinical characteristics. Study participants had a median age of 31 years, a median gravidity of 2 and parity of 1. 33.3% delivered via caesarean section. The median gestational age for GBS screening was 36 weeks. At presentation for delivery at the obstetric department, GBS screening results were available in 91.8% (901/982) of cases, for the remaining cases the result was available only later. A repeated GBS screening at the time of presentation in the hospital was performed in 155 PW; of these, 149 (96.1%) had a second positive screening result.

**Table 1:** Clinical characteristics of the study population

Variable		Total number of participants (n)	Results of variable
Maternal age at delivery in years	Median (interquartile range)	1081	31 (27–35)
Gravidity	Median (interquartile range)	971	2 (1–2)
Parity	Median (interquartile range)	973	1 (1–2)
Mode of delivery Vaginal delivery Vaginal-operative delivery Elective caesarean delivery Non-elective caesarean delivery	Absolute numbers (percentage)	884	529 (59.8%) 61 (6.9%) 144 (16.3%) 150 (17.0%)
GA at GBS screening in weeks	Median (interquartile range)	962	36 (35–37)

Different denominators due to missing variables

*GA* gestational age, *GBS* group B streptococcus

### Prenatal screening

Of the PW recorded as carriers of GBS, 79.9% (762/954) had a positive recto-vaginal culture for GBS and 20.1% (192/954) had a positive vaginal culture. The distribution of capsular serotypes did not significantly differ between the two body sites (data not shown). Positive GBS culture results were obtained using one, or a combination, of the different recommended selective agar media: Granada agar in 43.8% (474/1,083); blood agar with colistin and nalidixic acid (CNA) in 43.4% (470/1,083); StrepBSelect^™^ in 18.6% (201/1,083) and ChromID Strepto B^™^ in 8.3% (90/1,083), respectively.

### Intrapartum antibiotic prophylaxis and risk factors

In total, 77.8% (769/988) of PW received any IAP. For 683 of these PW, information on the given antimicrobial agent was available: either ampicillin or penicillin in 74.8% (511/683), followed by cephalosporins (112/683, 16.4%; Table 2). The median number of dosages prior to delivery was 2 and the first dose was administered with an average of 7.4 h prior to delivery (Table 2). 58.3% (382/655) of women received IAP with two doses prior to delivery. An appropriate IAP regarding choice of antibiotic, intravenous application, and number of doses was given to 49.5% (368/743) of PW. Of note, the frequency of antibiotic prophylaxis was significantly lower in PW giving birth to children with EOD, but rates were significantly higher in PW with other risk factors, in addition to positive GBS carrier status (Fig. 1a). Regarding IAP in the different participating countries, the range was widely varying from almost 100% (Spain) to below 40% (Bulgaria, supplemental Fig 1). In total, 13.4% (145/1,083) of all PW had at least one obstetrical risk factor for EOD besides GBS colonization, i.e. rupture of membranes of ≥ 18 h, intrapartum fever, or positive GBS urine culture as an indicator for heavy colonization (Fig. 1b).

**Table 2:** Intrapartum antibiotic prophylaxis

Variable		Total number of participants (n)	Results of variable
Intrapartum antibiotics	Total (percentage)	988	769 (77.8%)
Selection of maternal antibiotics Ampicillin Penicillin Cephalosporins Erythromycin Clindamycin Others	Total (percentage)	683	389 (60.0%) 122 (17.9%) 112 (16.4%) 17 (2.5%) 7 (1.0%) 18 (2.6%)
Indication for maternal antibiotics GBS prophylaxis Perioperative prophylaxis (Caesarean delivery) Suspected chorioamnionitis	Total (percentage)	751	679 (90.4%) 40 (5.3%) 2 (0.3%)
Route of maternal antibiotic administration Intravenous Oral	Total (percentage)	683	655 (95.6%) 28 (4.1%)
Hours of first antibiotic dose prior to delivery	Mean ± SD	650	7.38 ± 6.80
Total number of antibiotic dosages prior to delivery	Median	659	2

Different denominators due to missing variables

*GBS* group B streptococcus, *SD* standard deviation

### Early-onset neonatal GBS disease

Of 988 infants born to colonized women, nine developed GBS early onset disease (EOD). Due to the heterogenous distribution of EOD cases across Europe, with most countries reporting zero cases in the respective cohorts, estimating overall incidence rates from our data would not be meaningful. Seven cases were reported from the Czech Republic and two cases from Germany. Table 3 lists the characteristics of nine mother-infant pairs with EOD. Five of those patients were delivered vaginally, and four by emergency caesarean section. Four women had PROM ≥ 18 h and two developed intrapartum fever ≥ 38 °C, and five had at least one risk factor (Fig. 1b). In three cases, the GBS culture result was not known at the time of delivery. The localisation of the culture was vaginal in five patients and only two had an appropriate recto-vaginal culture. Eight out of the nine women did not receive appropriate IAP prior to delivery, while one PW received three doses of clindamycin, which is considered appropriate IAP despite potential antibiotic resistance (Table 3, Fig. 1a).

**Table 3:** Clinical characteristics and clinical GBS screening data of the nine mother-infant pairs with EOD

Mother-infant pair	Maternal age at delivery (years)	GA at screening timepoint	Localization of GBS screening	Duration of ROM before delivery (hours)	Intra-partum fever	Mode of delivery	IAP	Total antibiotic dosages prior to delivery	Sero-type
1	28	Unknown	Vaginal	> 18	Yes	Vaginal	Clinda-mycin	3	II
2	30	35	Vaginal	< 12 h	No	Vaginal	No	–	II
3	25	35	Vaginal	< 12	Yes	Vaginal	Peni-cillin	1	III
4	30	34	Vaginal	> 18	No	Vaginal	Spira-mycin	5	V
5	38	No screening result at delivery	Unknown	> 18	No	Emergency C-section	Azithro-mycin	1	II
6	32	36	Vaginal	12–18	No	Vaginal	No	–	V
7	25	No screening result at delivery	Unknown	> 18	No	Emergency C-section	Azithro-mycin	3	IV
8	36	35	Recto-vaginal	Unknown	No	Emergency C-section	No	–	II
9	32	No screening result at delivery	Recto-vaginal	Unknown	Un-known	Emergency C-section	No	–	Ia

*EOD* early-onset disease, *ROM* rupture of membranes, *GA* gestational age, *GBS* group B streptococcus, *IAP* intrapartum antibiotic prophylaxis

### Microbiological characteristics of isolated strains

Capsular serotyping of all 1083 GBS isolates was performed. 112 (10.3%) of isolates were non-typable by capsular serotyping, therefore capsular genotyping was performed for all isolates, here 54 isolates (5.0%) remained untypeable. Combining the two methods, we were able to determine the capsular type of all but six isolates (0.5%). Figure 2a displays the results from capsular serotyping, unless the isolate was untypeable, in that case the result from capsular genotyping is utilized. Of note, 26 isolates (2.4%) yielded discordant results between the two methods, in these cases the capsular serotype is used for further analysis (supplemental Table 4). We found no predominance of a specific combination, thus, while both methods are potentially erroneous, a systematic error in the two detection methods may be excluded. The predominant capsular serotype was type III (33.6%) followed by type Ia (20.2%), type V (18.4%), and type II (13.9%). Comparing capsular serotype prevalences across participating countries revealed some heterogeneity, e.g. an underrepresentation of CPS type IV in Germany. While type III was the most common isolate in every country, the rates of type Ia ranged from 3.5% in Bulgaria to 22.5% in the UK and for type V, it ranged from 7.3% in Denmark to 19.8% in Belgium (supplemental Table 1). No significant association between capsular serotype and age groups or between capsular serotype and parity status was observed (supplemental Tables 2 and 3).

Multilocus sequence types (MLST, Fig. 2b) of 669 GBS isolates were generated and clonal complexes (CC, Fig. 2c) were assigned to 666 isolates, the remaining three STs were singletons. The most common MLST sequence types were ST 19 (16.6%), ST 23 (16.0%), and ST 1 (14.9%). Clonal complex 19 (23.6%) was most frequently identified, followed by CC 1 (19.7%) and CC 23 (19.1%).

Pilus genes were detected in in 1069 out of 1083 isolates. A predominance of a type 1 + 2a pattern was observed (57.4%; Fig. 3a). GBS pilus analysis by serological procedures failed to demonstrate pilus expression in 215 isolates (19.9%), including the 14 strains lacking evidence of pilus genes. Another 40 isolates (3.7%) were not typeable. Among the 828 positive isolates, type 2a was the most prevalent expression type (51%, i.e. 39% of all isolates, Fig. 3b).

## Discussion

Despite the progress in knowledge about prevention of EOD and implementation of preventive strategies, GBS remains a major cause of neonatal morbidity and mortality. One of the aims of the European DEVANI project, studying more than 1,000 GBS-colonized pregnant women, was to assess European GBS epidemiology in order to facilitate the design of GBS vaccines [5, 16, 17]. In Europe, the majority of countries, except Bulgaria, Denmark, Greece, Norway, and the United Kingdom, offer universal prenatal screening for GBS colonization between 35 and 37 weeks of gestation and IAP in case of positive GBS screening. Since recently, a PCR-based screening method is increasingly used [30].

The median GA at screening in our cohort was 36 weeks, which is in accordance with the recommended screening period of 35–37 weeks. For better coverage up to 41 0/7 weeks of gestation, the American College of Obstetricians and Gynecologists (ACOG) committee recommends a new timing of universal GBS screening between 36 0/7 and 37 6/7 weeks of gestation [27]. In our cohort, 155 PW received a second screening when presenting for delivery; of these, 149 (96.1%) had a second positive screening result. While a similarly high percentage has been described before [31], this is an unusually high detection rate, consistent with a stable GBS carrier state. Only 13% of women had at least one additional obstetrical risk factor for EOD, i.e., the majority of colonized women would have been missed by a risk-factor based IAP approach, while all were at risk of giving birth to a neonate with EOD. In a large cohort of neonatal GBS sepsis, 66% of mothers did not have any additional risk factors, underlining the safer coverage of a universal screening approach as opposed to a risk factor-based approach [5]. The main risk factors for EOD are PROM ≥ 18 h and intrapartum fever [2, 9], which our study confirmed. A previously published meta-analysis found a risk of EOD of 1.1% (95%-confidence interval [CI] 0.6–1.5%) in settings without IAP policy and a risk of 0.03% (95%-CI 0–0.7%) with universal screening and a mean IAP coverage rate of 75% [14]. In the Czech Republic, 7 EOD cases were observed among 107 PW included in this study, in Germany 2 EOD cases were observed among 363 PW included. All other participating countries reported zero EOD cases. This regional difference could reflect regional differences in the management of the newborns, i.e. indication for antibiotic therapy in suspected infections without culture positivity. In addition, reporting bias could have influenced this regional heterogeneity.

In our study cohort, some kind of IAP was initiated in 77.8% (769/988) of cases, which is lower than the 89–93% in most published studies on screening-based IAP approaches [2, 14, 32]. This is in part attributable to 8.2% (81/982) of PW for whom screening results became available only after delivery. In addition, important regional differences were observed (Spain: almost 100%, Bulgaria: 40% IAP rate). An appropriate IAP regarding drug choice, application route, and treatment time was observed in only 49.5% (368/743) of our colonized PW. A substantial part of PW only present to the delivery ward with active labour status, thus the timespan is frequently too short for appropriate IAP [33]. In mothers with additional risk factors, IAP rates were higher, indicating that individual decisions influenced treatment choices. Risk factors were unevenly distributed across Europe, with GBS bacteriuria ranging from zero cases in several countries to 27.3% in Spain. Similarly, several countries reported zero PROM cases in our cohort, while Spain reported it in 13.4% of cases. Appropriate IAP was not administered to 8 out of 9 EOD cases, indirectly confirming the efficacy of IAP. Similarly, regarding the anatomical site of the screening, 20% of the cultures were vaginal and not vaginal-rectal or vaginal-perianal, which yield substantially better culture results [8, 27, 34]. Together, this reveals a low adherence to existing recommendations and to the study protocol even under study conditions and emphasizes the need for universal recommendations and continued efforts to maintain awareness for guideline adherence among health professionals.

While IAP is crucial for preventing group B streptococcus infections, its effects on the neonatal microbiome warrant careful consideration. It may pose risks to the neonate, particularly by disrupting the development of their microbiome. This disruption may lead to altered gut microbiota, potentially increasing susceptibility to infections and inflammatory conditions with possible long-term health consequences. These effects warrant consideration, when balancing the risks and benefits against the considerable rate of EOD in infants born to mothers without any additional risk factors.

A previously published systematic review found five capsular serotypes (i.e., Ia, Ib, II, III, V) to account for more than 85% of circulating capsular serotypes worldwide and in Europe these numbers increased to 96.1% in infants with EOD or LOD and 88% in carriers [3, 5]. As previously described, the high prevalence of pilus type 1 + 2a in colonizing isolates matches the higher frequencies of capsular serotype V, II and Ib in the colonization group compared to invasive GBS strains [16]. Our data shows that the same five capsular serotypes accounted for 92.6% of typeable colonizing GBS strains. Using a hexavalent vaccine [25] increases coverage to 98% of infants [5] and 96.6% of colonized PW, supporting this approach also for the European population. Indeed, given the repeatedly documented limitations of all screening procedures, the high disease burden of GBS in neonates and the promising results of vaccine trials [25], increased efforts are warranted to implement a universal GBS vaccine to pregnant women.

There are some limitations to our study. Prematurity as a risk factor was not assessed. Despite the eligibility criteria stating otherwise, 10 PW with an age below 18 years were included in the study and those cases were included in the analyses. Regional differences of GBS policies, including different IAP application standards make comparison of data difficult and partially explain the variations we have found. There is a number of missing variables in our data that could not be obtained in retrospect. However, the overall large number of cases may compensate for the missing variables. Lastly, the delay in reporting is attributable to difficulties in management of the dataset with missing variables from different countries, as well as to changes in personal responsibilities within the consortium. However, the authors strongly believe that the clinical and microbiological findings in our highly unique European cohort are still relevant to the current situation and reflect the spectrum of GBS disease nowadays.

In conclusion, continuous efforts to increase awareness and standardize compulsory screening procedures across Europe are required to decrease the disease burden of GBS in neonates. The World Health Organization (WHO) has identified the development of a GBS vaccine as a priority [35]. Our study provides detailed insight into the European prevalence of GBS capsular serotypes and pilus types, which is of crucial importance for future preventive strategies and vaccine development.

## Supplementary Information

Below is the link to the electronic supplementary material.

Supplementary file1 (PDF 1058 KB)

Supplementary file2 (DOCX 40 KB)

## Data Availability

Data is provided within the manuscript or supplementary information files.

## Abbreviations

ACOG: American College of Obstetricians and Gynecologists
CDC: Centers for Disease Control and Prevention
CPS: Capsular polysaccharide
CSF: Cerebrospinal fluid
GBS: Group B streptococcus, (GBS, species Streptococcus agalactiae)
DEVANI: Design of a Vaccine Against Neonatal Infections
EOD: Early-onset disease
IAP: Intrapartum antibiotic prophylaxis
LOD: Late-onset disease
MLST: Multi-locus sequence typing
PCR: Polymerase chain reaction
PROM: Preterm rupture of membranes, rupture of membranes before onset of labour
PW: Pregnant women
RCOG: Royal College of Obstetricians and Gynaecologists
UK: United Kingdom
WHO: World health organization

## References

[1] Melin P, Efstratiou A. Group B streptococcal epidemiology and vaccine needs in developed countries. Vaccine. 2013;31:D31-42. 10.1016/j.vaccine.2013.05.012. 23973345 10.1016/j.vaccine.2013.05.012

[2] Schrag SJ, Zywicki S, Farley MM, Reingold AL, Harrison LH, Lefkowitz LB, Hadler JL, Danila R, Cieslak PR, Schuchat A. Group B streptococcal disease in the era of intrapartum antibiotic prophylaxis. N Engl J Med. 2000;342:15–20. 10.1056/NEJM200001063420103. 10620644 10.1056/NEJM200001063420103

[3] Edmond KM, Kortsalioudaki C, Scott S, Schrag SJ, Zaidi AKM, Cousens S, Heath PT. Group B streptococcal disease in infants aged younger than 3 months: systematic review and meta-analysis. Lancet. 2012;379:547–56. 10.1016/S0140-6736(11)61651-6. 22226047 10.1016/S0140-6736(11)61651-6

[4] Puopolo KM, Lynfield R, Cummings JJ, Hand I, Adams-Chapman I, Poindexter B, Stewart DL, Aucott SW, Goldsmith JP, Mowitz M, Watterberg K, Maldonado YA, Zaoutis TE, Banerjee R, Barnett ED, Campbell JD, Gerber JS, Kourtis AP, Munoz FM, Nolt D, Nyquist AC, O’Leary ST, Sawyer MH, Steinbach WJ, Zangwill K, Committee on fetus and newborn, Committee on infectious diseases. Management of infants at risk for group B Streptococcal disease. Pediatrics. 2019;144:e20191881. 10.1542/peds.2019-1881. 31285392 10.1542/peds.2019-1881

[5] Lohrmann F, Hufnagel M, Kunze M, Afshar B, Creti R, Detcheva A, Kozakova J, Rodriguez-Granger J, Sørensen UBS, Margarit I, Maione D, Rinaudo D, Orefici G, Telford J, de la Rosa FM, Kilian M, Efstratiou A, Berner R, Melin P. Neonatal invasive disease caused by *Streptococcus* agalactiae in Europe: the DEVANI multi-center study. Infection. 2023;51:981–91. 10.1007/s15010-022-01965-x. 36547864 10.1007/s15010-022-01965-x PMC9773664

[6] Schrag SJ, Verani J. Intrapartum antibiotic prophylaxis for the prevention of perinatal group B streptococcal disease: experience in the United States and implications for a potential group B streptococcal vaccine. Vaccine. 2013. 10.1016/j.vaccine.2012.11.056. 23219695 10.1016/j.vaccine.2012.11.056 PMC11843781

[7] ACOG (American Congress of Obstetricians and Gynecologists) Committee on Obstetric Practice. ACOG committee opinion no 485: prevention of early-onset group B streptococcal disease in newborns. Obstet Gynecol. 2011;117:1019–27. 10.1097/AOG.0b013e318219229b. PMID: 31977793. 21422882 10.1097/AOG.0b013e318219229b

[8] Verani M JR, L S, S.J. (2010) Division of bacterial diseases, national center for immunization and respiratory diseases, centers for disease control and prevention (CDC. Prevention of perinatal group B streptococcal disease–revised guidelines from CDC 59:1–36

[9] Schrag S, Gorwitz R, Fultz-Butts K, Schuchat A. Prevention of perinatal group B streptococcal disease. Revis Guidel CDC Morb Mort Wkly Rep Recomm Rep. 2002;51:1–22. 12211284

[10] Hughes RG, Brocklehurst P, Steer PJ, Heath P. Stenson BM on behalf of the royal college of obstetricians and gynaecologists prevention of early-onset neonatal group B streptococcal disease. Green-top Guidel. 2017. 10.1111/1471-0528.14821.

[11] Kunze M, Ziegler A, Fluegge K, Hentschel R, Proempeler H, Berner R. Colonization, serotypes and transmission rates of group B streptococci in pregnant women and their infants born at a single university center in Germany. J Perinat Med. 2011;39:417–22. 10.1515/jpm.2011.037. 21557677 10.1515/jpm.2011.037

[12] Rodriguez-Granger J, Alvargonzalez JC, Berardi A, Berner R, Kunze M, Hufnagel M, Melin P, Decheva A, Orefici G, Poyart C, Telford J, Efstratiou A, Killian M, Krizova P, Baldassarri L, Spellerberg B, Puertas A, Rosa-Fraile M. Prevention of group B streptococcal neonatal disease revisited. The DEVANI European project. Eur J Clin Microbiol Infect Dis. 2012;31:2097–104. 10.1007/s10096-012-1559-0. 22314410 10.1007/s10096-012-1559-0

[13] Bekker V, Bijlsma MW, van de Beek D, Kuijpers TW, van der Ende A. Incidence of invasive group B streptococcal disease and pathogen genotype distribution in newborn babies in the Netherlands over 25 years: a nationwide surveillance study. Lancet Infect Dis. 2014;14:1083–9. 10.1016/S1473-3099(14)70919-3. 25444407 10.1016/S1473-3099(14)70919-3

[14] Russell NJ, Seale AC, O’Sullivan C, Le Doare K, Heath PT, Lawn JE, Bartlett L, Cutland C, Gravett M, Ip M, Madhi SA, Rubens CE, Saha SK, Schrag S, Sobanjo-Ter Meulen A, Vekemans J, Baker CJ. Risk of early-onset neonatal group b streptococcal disease with maternal colonization worldwide systematic review meta-analyses. Clin Infect Dis. 2017;65:S152–9. 10.1093/cid/cix655. 29117325 10.1093/cid/cix655 PMC5850448

[15] Rodriguez-Granger J, Alvargonzalez JC, Berardi A, Berner R, Kunze M, Hufnagel M, Melin P, Decheva A, Orefici G, Poyart C, Telford J, Efstratiou A, Killian M, Krizova P, Baldassarri L, Spellerberg B, Puertas A, Rosa-Fraile M. Prevention of group B streptococcal neonatal disease revisited DEVANI European project. Eur J Clin Microbiol Infect Dis. 2012;31:2097–104. 10.1007/s10096-012-1559-0. 22314410 10.1007/s10096-012-1559-0

[16] Fabbrini M, Rigat F, Rinaudo CD, Passalaqua I, Khacheh S, Creti R, Baldassarri L, Carboni F, Anderloni G, Rosini R, Maione D, Grandi G, Telford JL, Margarit I. The protective value of maternal group B *Streptococcus* antibodies: quantitative and functional analysis of naturally acquired responses to capsular polysaccharides and pilus proteins in European maternal sera. Clin Infect Dis. 2016;63:746–53. 10.1093/cid/ciw377. 27402816 10.1093/cid/ciw377

[17] Afshar B, Broughton K, Creti R, Decheva A, Hufnagel M, Kriz P, Lambertsen L, Lovgren M, Melin P, Orefici G, Poyart C, Radtke A, Rodriguez-Granger J, Sørensen UBS, Telford J, Valinsky L, Zachariadou L, Efstratiou A, Members of the DEVANI Study Group. International external quality assurance for laboratory identification and typing of Streptococcus agalactiae (group B streptococci). J Clin Microbiol. 2011;49:1475–82. 21325542 10.1128/JCM.02365-10 PMC3122801

[18] Yao K, Poulsen K, Maione D, Rinaudo CD, Baldassarri L, Telford JL, Sørensen UBS, Kilian M, Members of the DEVANI Study Group. Capsular gene typing of Streptococcus agalactiae compared to serotyping by latex agglutination. J Clin Microbiol. 2013;51:503–7. 10.1128/JCM.02417-12. 23196363 10.1128/JCM.02417-12 PMC3553911

[19] Rosini R, Campisi E, De Chiara M, Tettelin H, Rinaudo D, Toniolo C, Metruccio M, Guidotti S, Sørensen UBS, Kilian M, Ramirez M, Janulczyk R, Donati C, Grandi G, Margarit I, DEVANI Consortium. Genomic analysis reveals the molecular basis for capsule loss in the group B Streptococcus population. PLoS ONE. 2015;10:e0125985. 10.1371/journal.pone.0125985. 25946017 10.1371/journal.pone.0125985 PMC4422693

[20] Lohrmann F, Berg A, Wicker E, Imm A, Krause G, Zürn K, Berner R, Hufnagel M, Lander F. Prevalence of capsular serotype, Pilus Island distribution, and antibiotic resistance in pediatric and adult invasive Group B Streptococcus isolates: data from a nationwide prospective surveillance study in Germany. Pediatr Infect Dis J. 2021;40:76–82. 10.1097/INF.0000000000002943. 33201062 10.1097/INF.0000000000002943

[21] Creti R, Imperi M, Berardi A, Pataracchia M, Recchia S, Alfarone G, Baldassarri L, Italian Neonatal GBS Infections Working Group. Neonatal group B streptococcus infections: prevention strategies, clinical and microbiologic characteristics in 7 years of surveillance. Pediatr Infect Dis J. 2017;36:256–62. 10.1097/INF.0000000000001414. 27870810 10.1097/INF.0000000000001414

[22] Absalon J, Segall N, Block SL, Center KJ, Scully IL, Giardina PC, Peterson J, Watson WJ, Gruber WC, Jansen KU, Peng Y, Munson S, Pavliakova D, Scott DA, Anderson AS. Safety and immunogenicity of a novel hexavalent group B streptococcus conjugate vaccine in healthy, non-pregnant adults: a phase 1/2, randomised, placebo-controlled, observer-blinded, dose-escalation trial. Lancet Infect Dis. 2021;21:263–74. 10.1016/S1473-3099(20)30478-3. 32891191 10.1016/S1473-3099(20)30478-3 PMC9760110

[23] Baker CJ, Rench MA, McInnes P. Immunization of pregnant women with group B streptococcal type III capsular polysaccharide-tetanus toxoid conjugate vaccine. Vaccine. 2003;21:3468–72. 10.1016/s0264-410x(03)00353-0. 12850362 10.1016/s0264-410x(03)00353-0

[24] Swamy GK, Metz TD, Edwards KM, Soper DE, Beigi RH, Campbell JD, Grassano L, Buffi G, Dreisbach A, Margarit I, Karsten A, Henry O, Lattanzi M, Bebia Z. Safety and immunogenicity of an investigational maternal trivalent group B streptococcus vaccine in pregnant women and their infants: results from a randomized placebo-controlled phase II trial. Vaccine. 2020;38:6930–40. 10.1016/j.vaccine.2020.08.056. 32883555 10.1016/j.vaccine.2020.08.056

[25] Madhi SA, Anderson AS, Absalon J, Radley D, Simon R, Jongihlati B, Strehlau R, van Niekerk AM, Izu A, Naidoo N, Kwatra G, Ramsamy Y, Said M, Jones S, Jose L, Fairlie L, Barnabas SL, Newton R, Munson S, Jefferies Z, Pavliakova D, Silmon de Monerri NC, Gomme E, Perez JL, Scott DA, Gruber WC, Jansen KU. Potential for maternally administered vaccine for infant group B streptococcus. N Engl J Med. 2023;389:215–27. 10.1056/NEJMoa2116045. 37467497 10.1056/NEJMoa2116045

[26] Imperi M, Pataracchia M, Alfarone G, Baldassarri L, Orefici G, Creti R. A multiplex PCR assay for the direct identification of the capsular type (Ia to IX) of Streptococcus agalactiae. J Microbiol Methods. 2010;80:212–4. 10.1016/j.mimet.2009.11.010. 19958797 10.1016/j.mimet.2009.11.010

[27] Prevention of group B streptococcal early-onset disease in newborns: ACOG committee opinion summary, Number 797. Obstet Gynecol. 2020;135(2):489–492. 10.1097/AOG.0000000000003669. 10.1097/AOG.0000000000003669 31977793

[28] Madrid L, Seale AC, Kohli-Lynch M, Edmond KM, Lawn JE, Heath PT, Madhi SA, Baker CJ, Bartlett L, Cutland C, Gravett MG, Ip M, Le Doare K, Rubens CE, Saha SK, Sobanjo-Ter Meulen A, Vekemans J, Schrag S, Infant GBS Disease Investigator Group. Infant group B streptococcal disease incidence and serotypes worldwide: systematic review and meta-analyses. Clin Infect Dis. 2017;65:S172. 10.1093/cid/cix656. 10.1093/cid/cix656 PMC5850457 29117326

[29] R Core Team (2024) R: A Language and Environment for Statistical Computing. R Foundation for Statistical Computing, Vienna, Austria

[30] Vieira LL, Perez AV, Machado MM, Kayser ML, Vettori DV, Alegretti AP, Ferreira CF, Vettorazzi J, Valério EG. Group B Streptococcus detection in pregnant women: comparison of qPCR assay, culture, and the Xpert GBS rapid test. BMC Pregnancy Childbirth. 2019;19:532. 10.1186/s12884-019-2681-0. 31888631 10.1186/s12884-019-2681-0 PMC6937909

[31] Hansen SM, Uldbjerg N, Kilian M, Sørensen UBS. Dynamics of Streptococcus agalactiae colonization in women during and after pregnancy and in their infants. J Clin Microbiol. 2004;42:83–9. 10.1128/JCM.42.1.83-89.2004. 14715736 10.1128/JCM.42.1.83-89.2004 PMC321715

[32] Kunze M, Zumstein K, Markfeld-Erol F, Elling R, Lander F, Prömpeler H, Berner R, Hufnagel M. Comparison of pre- and intrapartum screening of group B streptococci and adherence to screening guidelines: a cohort study. Eur J Pediatr. 2015;174:827–35. 10.1007/s00431-015-2548-y. 25922140 10.1007/s00431-015-2548-y

[33] Kjerulff KH, Attanasio LB, Vanderlaan J, Sznajder KK. Timing of hospital admission at first childbirth: A prospective cohort study. PLoS ONE. 2023;18:e0281707. 10.1371/journal.pone.0281707. 36795737 10.1371/journal.pone.0281707 PMC9934383

[34] Williams M, Zantow E, Turrentine M. Cost effectiveness of latest recommendations for group B streptococci screening in the United States. Obstet Gynecol. 2020;135:789–98. 32168204 10.1097/AOG.0000000000003649

[35] WHO Group B Streptococcus (GBS). https://www.who.int/teams/immunization-vaccines-and-biologicals/diseases/group-b-streptococcus-(gbs). Accessed 11 Jul 2024

