# Supplementary material for: Maternal Streptococcus agalactiae colonization in Europe: data from the multi-center DEVANI study
Source: Infection. 2024 Sep 8;53(1):373–81. doi: 10.1007/s15010-024-02380-0 (PMC11825526; doi:10.1007/s15010-024-02380-0)
Supplement: Supplementary file 1 — Supplementary file1 (PDF 1058 KB) [file 15010_2024_2380_MOESM1_ESM.pdf]

Supplemental table 1 - Serotypes across countries

Country	**Ia**	**Ib**	**II**	**III**	**IV**	**V**	**VI**	**VII**	**VIII**	**IX**	**Nt**
Absolute numbers											
Belgium	20	7	15	31	11	26	1	0	0	0	0
Bulgaria	5	2	10	8	1	3	0	0	0	0	0
Czech Republic	18	4	18	30	3	28	2	3	0	1	0
Denmark	16	7	14	23	5	11	0	0	0	5	1
Germany	73	27	44	136	2	65	1	3	0	9	3
Italy	16	2	12	38	4	17	0	0	0	3	1
Spain	42	7	28	57	10	31	0	0	1	11	0
United Kingdom	29	9	10	41	2	18	1	0	0	0	1
Percentage											
Belgium	18%	6.3%	13.5%	27.9%	9.9%	23.4%	0.9%	0%	0%	0%	0%
Bulgaria	17.2%	6.9%	34.5%	27.6%	3.4%	10.3%	0%	0%	0%	0%	0%
Czech Republic	16.8%	3.7%	16.8%	28%	2.8%	26.2%	1.9%	2.8%	0%	0.9%	0%
Denmark	19.5%	8.5%	17.1%	28%	6.1%	13.4%	0%	0%	0%	6.1%	1.2%
Germany	20.1%	7.4%	12.1%	37.5%	0.6%	17.9%	0.3%	0.8%	0%	2.5%	0.8%
Italy	17.2%	2.2%	12.9%	40.9%	4.3%	18.3%	0%	0%	0%	3.2%	1.1%
Spain	22.5%	3.7%	15%	30.5%	5.3%	16.6%	0%	0%	0.5%	5.9%	0%
United Kingdom	26.1%	8.1%	9%	36.9%	1.8%	16.2%	0.9%	0%	0%	0%	0.9%
Raw p-value											
Belgium	0.627	1	1	0.218	**0**	0.187	0.418	1	1	0.064	1
Bulgaria	0.864	0.691	**0.003**	0.619	1	0.336	1	1	1	1	1
Czech Republic	0.426	0.393	0.448	0.239	1	**0.039**	0.079	**0.015**	1	0.351	1
Denmark	0.981	0.445	0.493	0.323	0.311	0.29	1	1	1	0.101	0.377
Germany	1	0.201	0.256	0.066	**0**	0.842	0.669	0.409	1	0.93	0.409
Italy	0.533	0.113	0.884	0.152	0.561	1	1	1	1	0.732	0.417
Spain	0.461	0.208	0.74	0.362	0.199	0.553	0.594	0.597	0.173	**0.006**	0.597
United Kingdom	0.131	0.438	0.15	0.498	0.419	0.624	0.418	1	1	0.064	0.478

Supplemental table 1 - Serotypes across countries

Country	**Ia**	**Ib**	**II**	**III**	**IV**	**V**	**VI**	**VII**	**VIII**	**IX**	**Nt**
Adjusted p-value											
Belgium	1	1	1	0.914	**0.014**	0.913	0.961	1	1	0.645	1
Bulgaria	1	1	0.089	1	1	0.961	1	1	1	1	1
Czech Republic	0.961	0.961	0.961	0.956	1	0.576	0.698	0.265	1	0.961	1
Denmark	1	0.961	0.975	0.961	0.961	0.961	1	1	1	0.809	0.961
Germany	1	0.913	0.961	0.645	**0.003**	1	1	0.961	1	1	0.961
Italy	1	0.828	1	0.89	1	1	1	1	1	1	0.961
Spain	0.965	0.913	1	0.961	0.913	1	1	1	0.913	0.137	1
United Kingdom	0.887	0.961	0.89	0.975	0.961	1	0.961	1	1	0.645	0.975

Supplemental table 2 - Serotypes across age groups

Age group	**Ia**	**Ib**	**II**	**III**	**IV**	**V**	**VI**	**VII**	**VIII**	**IX**	**Nt**
Absolute numbers											
<20	6	3	7	7	2	5	0	0	0	1	0
20-25	30	10	22	48	7	19	1	2	0	2	1
26-30	70	16	42	110	12	66	2	1	1	4	2
31-35	73	23	51	126	7	72	1	3	0	14	2
>35	40	13	29	71	10	37	1	0	0	8	1
Percentage											
<20	19.4%	9.7%	22.6%	22.6%	6.5%	16.1%	0%	0%	0%	3.2%	0%
20-25	21.1%	7%	15.5%	33.8%	4.9%	13.4%	0.7%	1.4%	0%	1.4%	0.7%
26-30	21.5%	4.9%	12.9%	33.7%	3.7%	20.2%	0.6%	0.3%	0.3%	1.2%	0.6%
31-35	19.6%	6.2%	13.7%	33.9%	1.9%	19.4%	0.3%	0.8%	0%	3.8%	0.5%
>35	19%	6.2%	13.8%	33.8%	4.8%	17.6%	0.5%	0%	0%	3.8%	0.5%
Raw p-value											
<20	1	0.427	0.254	0.266	0.298	0.923	1	1	1	0.575	1
20-25	0.87	0.716	0.665	1	0.461	0.123	0.506	0.18	1	0.413	0.571
26-30	0.569	0.387	0.561	0.963	0.988	0.348	0.641	0.674	0.302	0.063	1
31-35	0.767	0.972	0.932	0.9	0.053	0.618	0.665	0.42	1	0.163	1
>35	0.696	1	1	0.977	0.377	0.818	1	0.603	1	0.375	1
Adjusted p-value											
<20	1	1	1	1	1	1	1	1	1	1	1
20-25	1	1	1	1	1	1	1	1	1	1	1
26-30	1	1	1	1	1	1	1	1	1	1	1
31-35	1	1	1	1	1	1	1	1	1	1	1
>35	1	1	1	1	1	1	1	1	1	1	1

Supplemental table 3 - Serotypes across parity status

Parity	**Ia**	**Ib**	**II**	**III**	**IV**	**V**	**VI**	**VII**	**VIII**	**IX**	**Nt**
Absolute numbers											
0	13	8	16	24	9	10	1	0	0	1	1
1	123	31	78	183	19	109	2	4	1	19	1
2	35	11	36	84	5	49	1	2	0	8	2
3	12	1	6	28	1	8	0	0	0	1	1
>3	7	5	5	4	2	5	0	0	0	0	0
Percentage											
0	15.7%	9.6%	19.3%	28.9%	10.8%	12%	1.2%	0%	0%	1.2%	1.2%
1	21.6%	5.4%	13.7%	32.1%	3.3%	19.1%	0.4%	0.7%	0.2%	3.3%	0.2%
2	15%	4.7%	15.5%	36.1%	2.1%	21%	0.4%	0.9%	0%	3.4%	0.9%
3	20.7%	1.7%	10.3%	48.3%	1.7%	13.8%	0%	0%	0%	1.7%	1.7%
>3	25%	17.9%	17.9%	14.3%	7.1%	17.9%	0%	0%	0%	0%	0%
Raw p-value											
0	0.43	0.181	0.259	0.453	**0.001**	0.144	0.301	1	1	0.504	0.361
1	0.069	0.708	0.439	0.414	0.578	0.693	1	1	1	0.567	0.166
2	0.057	0.535	0.717	0.333	0.213	0.324	1	0.634	1	0.809	0.345
3	0.956	0.247	0.462	**0.018**	0.718	0.424	1	1	1	1	0.265
>3	0.62	**0.018**	0.811	**0.04**	0.278	1	1	1	1	1	1
Adjusted p-value											
0	1	1	1	1	0.054	1	1	1	1	1	1
1	0.631	1	1	1	1	1	1	1	1	1	1
2	0.627	1	1	1	1	1	1	1	1	1	1
3	1	1	1	0.331	1	1	1	1	1	1	1
>3	1	0.331	1	0.546	1	1	1	1	1	1	1

Supplemental table 4 -
Discordance between capsular serotype and genotype

	Capsular genotype												
Capsular serotype	Ia	Ib	II	III	IV	V	VI	VII	VIII	IX	MIX II/VI	MIX III/V	Nt
Ia	0	1	0	2	0	0	0	0	0	0	0	0	9
Ib	0	0	0	0	0	0	0	0	0	0	0	0	2
II	0	0	0	0	0	1	2	0	0	0	0	0	3
III	0	0	3	0	1	3	1	0	0	0	1	1	22
IV	0	0	0	0	0	1	0	0	0	0	0	0	0
V	1	0	0	0	0	0	0	0	0	0	0	0	9
VI	0	0	0	0	0	0	0	0	0	0	0	0	0
VII	0	0	2	0	0	0	0	0	0	0	0	0	1
VIII	0	0	0	0	0	0	0	0	0	0	0	0	0
IX	1	0	3	0	2	0	0	0	0	0	0	0	0
Nt	28	7	21	21	5	22	0	0	0	2	0	0	0
