# Supplementary figures and images for: Maternal Streptococcus agalactiae colonization in Europe: data from the multi-center DEVANI study
Source: Infection. 2024 Sep 8;53(1):373–81. doi: 10.1007/s15010-024-02380-0 (PMC11825526; doi:10.1007/s15010-024-02380-0)

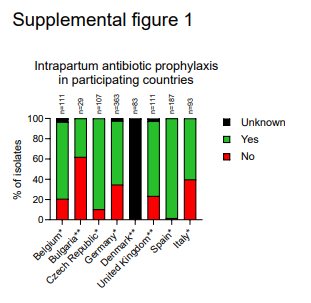

Supplement: Supplementary file 2 — Supplementary file2 (DOCX 40 KB) [file 15010_2024_2380_MOESM2_ESM.docx]
